# Gap junction blockers: a potential approach to attenuate morphine withdrawal symptoms

**DOI:** 10.1186/1423-0127-20-77

**Published:** 2013-10-21

**Authors:** Sabah Moradi, Mohammad Charkhpour, Hamed Ghavimi, Rasoul Motahari, Majid Ghaderi, Kambiz Hassanzadeh

**Affiliations:** 1 Department of Pharmacology and Toxicology, Faculty of Pharmacy, Tabriz University of Medical Sciences, Tabriz, Iran; 2 Student Research Committee, Tabriz University of Medical Sciences, Tabriz, Iran; 3 Department of Biology, Faculty of Science, Islamic Azad University, Sanandaj Branch, Sanandaj, Iran; 4 Cellular and Molecular Research Center, Kurdistan University of Medical Sciences, Sanandaj, Iran; 5 Department of Physiology and Pharmacology, Faculty of Medicine, Kurdistan University of Medical Sciences, Sanandaj, Iran

**Keywords:** Carbenoxolone, Mefloquine, Morphine, Withdrawal symptoms

## Abstract

**Background:**

The exact mechanisms of morphine-induced dependence and withdrawal symptoms remain unclear. In order to identify an agent that can prevent withdrawal syndrome, many studies have been performed. This study was aimed to evaluate the effect of gap junction blockers; carbenoxolone (CBX) or mefloquine (MFQ); on morphine withdrawal symptoms in male rat.

Adult male Wistar rats (225 – 275 g) were selected randomly and divided into 10 groups. All groups underwent stereotaxic surgery and in order to induce dependency, morphine was administered subcutaneously) Sc) at an interval of 12 hours for nine continuous days. On the ninth day of the experiment, animals received vehicle or CBX (100, 400, 600 μg/10 μl/rat, icv) or MFQ (50, 100 and 200 μg/10 μl/rat, icv) after the last saline or morphine (Sc) injection. Morphine withdrawal symptoms were precipitated by naloxone hydrochloride 10 min after the treatments. The withdrawal signs including: jumping, rearing, genital grooming, abdomen writhing, wet dog shake and stool weight, were recorded for 60 minutes.

**Results:**

Results showed that CBX and MFQ decreased all withdrawal **s**igns; and the analysis indicated that they could attenuate the total withdrawal scores significantly.

**Conclusion:**

Taking together it is concluded that gap junction blockers prevented naloxone-precipitated withdrawal symptoms.

## Background

It is well known that repeated administration of opiates results in physical dependence. This major side effect of opiates administration, limits their clinical application [1]. Dependence is a behavioral state requiring continued drug administration to avoid a series of aversive withdrawal symptoms. Therefore, new drugs and strategies are under investigation for preventing of opiate dependence as well as withdrawal signs in a wide variety of animal species. The neurotransmitter systems have been widely studied to find out the involved mechanisms of withdrawal symptoms. Several lines of evidence indicate the involvement of noradrenergic system in opiate withdrawal symptoms [2-4]. Although the factors and the brain regions or nucleolus involved in opiate dependence and withdrawal symptoms have been heavily investigated during two past decades. However the exact mechanisms of these phenomena are not completely understood.

The locus coeruleus (LC) area has been found to be the most sensitive site for the elicitation of motor aspects of opiate withdrawal [5,6]. It is a bilateral nucleus in the brainstem consisting mostly of noradrenergic neurons. Through a widespread efferent projection system, the locus coeruleus–noradrenergic (LC-NE) system supplies norepinephrine (NE) throughout the central nervous system [7]. During withdrawal of the opiates, the LC neurons exhibit an augmented activation of their noradrenergic discharge activity. Also there is growing evidence that gap junctions play an important role in the synchronization of neuronal oscillatory activity that has been implicated in many cognitive processes and in the generation of epileptic discharges [8].

Gap junctions are the channel-forming structures between the membranes of two abutting cells which allow direct electrical communication between cells [8]. Intercellular communication mediated by gap junction channels plays an important role in a variety of tissues, including the nervous system, lens, and heart, by allowing the passage of ions and small molecules between adjacent cells [9]. To date, the most thoroughly studied problem has been the involvement of gap junctions in seizure activity and the possibility of applying gap junction blockers to decrease epileptic discharges [10].

From the other side, carbenoxolone (CBX), a well-known gap junction inhibitor, could block the electrical coupling of neurons in LC therefore decreased synchronization of the spontaneous activity in this site [11].

CBX is a derivative of glycyrrhetinic acid, which has been used in the treatment of gastric and duodenal ulcers [12], directly binds to and blocks a broad spectrum of the connexins (Cx) that make up gap junctions or hemichannels [13,14].

In addition CBX could block the voltage-gated Ca^2+^ channels [15] and NMDA-evoked currents [16]. Furthermore, CBX is known to enhance the effects of endogenous glucocorticoid hormones by inhibiting 11beta-hydroxysteroid dehydrogenase [17].

Mefloquine, another potent gap junction blocker has been found to be relatively selective for certain subtypes of gap junctions [18]. Mefloquine has been commonly used in the prophylaxis and treatment of malaria and it could inhibit the IP_3_- induced Ca^2+^ release [19], inhibition of acetylcholinesterase activity [20], blockade of adenosine A_2A_ receptors [21] and inhibition of ATP-sensitive K channels [22].

Because of the similarity between withdrawal sings and the firings occur during the seizure and the role of gap junction inhibitors on preventing of epileptic discharges, in the present study we were interested to verify the effect of intracerebroventricular (icv) central administration of carbenoxolone and mefloquine as a gap junction blockers on morphine withdrawal symptoms.

## Methods

### Animals

Male Wistar rats (225-275 g) were purchased from the Pasteur Institute of Iran. They were housed six rats per cage (40 × 40 × 20 cm) at laboratory temperature (20 ± 3°C) and humidity (60%) under a 12-h light–dark cycle (lights on at 07:00 A.M). Food (lab chow) and water were available *ad libitum*. All procedures for animals were approved by the research committee of the Tabriz University of Medical Sciences and were performed according to the Guide for Care and Use of Laboratory Animals published by the United States National Institutes of Health (NIH Publication No. 85–23, revised 1985).

### Experimental groups

Rats were randomly divided into 10 groups:

1) One chronically subcutaneously (sc) normal saline solution (1 mL/kg, sc) and normal saline solution (10 μL/rat, icv) treated group (n = 8)

2) One chronically subcutaneously (sc) normal saline solution (1 mL/kg, sc) and DMSO 25% in saline (10 μL/rat, icv) treated group (n = 8)

3) One chronically subcutaneously (sc) Morphine and normal saline solution (10 μL/rat, icv) treated group (n = 8): Control for carbenoxolone

4) One chronically subcutaneously (sc) Morphine and DMSO 25% in saline (10 μL/rat, icv) treated group (n = 8): Control for mefloquine

5) Three chronically subcutaneously (sc) Morphine and CBX (100, 400, 600 μg/10ul/rat, icv) treated groups (n = 8)

6) Three chronically subcutaneously (sc) Morphine and MFQ (50, 100 and 200 μg/10 μl/rat, icv) treated groups (n = 8).

### Surgery and icv cannula implantation

Rats were anesthetized with a mixture of ketamin (100 mg/Kg, ip) and xylazine (5 mg/Kg, ip) and then placed on a stereotaxic apparatus and secured using blunt rodent ear bars. Lidocaine (2% V/V) was used for local anesthesia then the skull was surgically exposed and a stainless-steel guide cannula (3.4 mm long, 0.65 mm outer diameter, 23 gauge needle) was unilaterally implanted in to the injection site (coordinates for site of in injection: -0.8 mm posterior, -1.3 mm midline to lateral and 3.4 mm ventral according to the Rat Brain Atlas) [23]. The guide cannula tips were placed 1 mm above the injection site to minimize cellular damage. The guide cannula was fixed to the skull using two stainless-steel screws anchored to the skull and dental acrylic cement. Animals were allowed to recover by about 7 days after surgery [24].

### Drugs

The following drugs were used: Morphine sulfate and Naloxone naloxone hydrochloride (Darou Pakhsah Co. Iran), carbenoxolone (Sigma-Aldrich, St. Louis, USA), mefloquine (Sigma-Aldrich, St. Louis, USA), ketamin (Trittau, Germany) and xylazine (Alfasan, Netherland). Morphine, carbenoxolone and naloxone were dissolved in normal saline (0.9%) and MFQ dissolve in 25% dimethyl sulfoxide (DMSO) in saline before injection. Naloxone was injected to the all groups.

### Intracerebroventricular drug administration

Intracerebroventricular (icv) injection of CBX, MFQ, Saline and DMSO 25% were performed by removing the stylet from guide cannula and lowering a stainless steel injector cannula (14 mm, 30 gauge needle) that extended 1.0 mm beyond the implanted guide cannula tip. The injector cannula was connected to a 25-μl hamilton microsyringe by a polyethylene -20 (PE-20) tubing and 10 μl of CBX, MFQ or their vehicles were infused over 60s period. In order to minimize the drug back flow into the injection track, the cannula was gently withdrawn 60s after the injection and followed by replacement of the stylet. During the filling of injection system, a small air bubble was introduced into the PE-20 tubing to monitor the movement of the fluid during the injection.

### Induction of morphine dependence

In order to induce the dependency, additive doses of morphine were administered subcutaneously for nine days as follows: Day 1: 5 mg/kg /12 h, Day 2,3: 10 mg/kg/12 h, Day 4,5: 15 mg/kg /12 h, Day 6,7: 20 mg/kg/12 h, Day 8,9: 25 mg/kg/12 h. On the ninth day only the morning dose of morphine was injected then CBX or MFQ or their vehicles (saline or DMSO 25%, respectively) administered after 20 minutes. The method for additive doses of morphine was adapted from previous studies [25,26] which indicated that the dependency was established using this summing doses.

### Induction of morphine withdrawal

In order to induce withdrawal symptoms, naloxone (4 mg/kg, ip) was injected on the ninth day, 30 minutes after the morning dose of morphine injection (10 minutes after drugs or their vehicles injection). The chosen dose for naloxone was based on previous studies [25].

### Measurement of behavioral signs

Immediately after naloxone injection, the behavioral assessment of opioid withdrawal signs including jumping, rearing, genital grooming, abdomen writhing and wet-dog-shake was started for one hour. Animals were studied individually in a clear plexiglass chamber (50 × 25 × 15 cm) that was placed in other dark chamber to avoid environmental perturbations. A digital camera connected to a recording computer was placed on the inner chamber to simultaneously show the rats behaviors. The reactions of each animal were evaluated by an observer who was not aware of the nature of the treatment received by that animal. The behaviors of all animals were evaluated by the same observer.

In present study the total withdrawal score was calculated for each animal in addition to reporting the symptoms alone because the individual withdrawal signs under the various conditions appear different patterns of activity and there were significant differences in the specific behaviors exhibited by individual animals. Thus, the total withdrawal score measuring shows a general trend in the composite withdrawal scores which might be differed under different conditions [27].

To do this, 5 assessed signs in an animal were scored on the basis of the division of absolute frequency of each behavior into its respective weighting factor (Table 1). Then the scores of withdrawal signs were added to obtain a total withdrawal score (TWS) for each animal [24,27,28].

**Table 1:** Weighing factor of different withdrawal signs

**Behavior signs**	**Weighing factor**
Jumping	4
Wet dog shake	5
Abdomen writhing	5
Genital grooming	5
Rearing	20

### Locomotor activity test

Locomotor activity was assessed by direct observation using interval scale that measures the frequency of crossing the lines painted on the underside of floor of the Plexiglas behavioral arena by each animal. Locomotor activity test was done before and after icv injection [29].

### Histological verification of cannula placement

On completion of each experiment for evaluation the precise injecting site , methylene blue solution (5 μL/rat, icv) was injected to the cannula, and then animals were euthanized with pentobarbital followed by decapitation. The brain of each animal was dissected out and cut along the coronal plane to verify the placement of the guide cannula and the distribution of the methylene blue in the ventricles after the behavioral tests. Data for only those animals that displayed a uniform distribution of methylene blue in the ventricles were considered for statistical analysis. Overall, six animals were discarded because the placement of the guide cannula was incorrect.

### Data analysis

Statistical comparisons among the experimental groups were made by a one way analysis of variance (ANOVA) followed by Tukey’s post-hoc test for multiple comparisons. All results are shown as the mean ± SEM (standard error of the mean), with statistical significance set at p < 0.05.

## Results and discussion

### Naloxone-induced withdrawal

**Figure 1:**
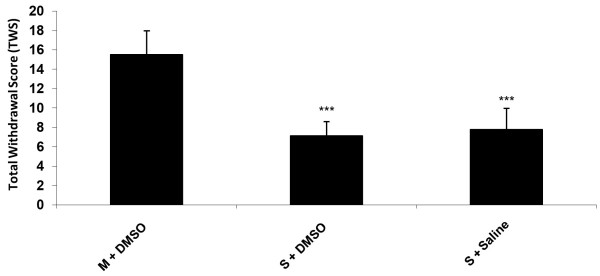
**Naloxone (4 mg/kg)-induced TWS in morphine-dependent rats in comparison to saline treated animals.** Data are expressed as mean ± S.E.M. ^***^p < 0.001 different from control (morphine-dependent DMSO 25% microinjected group). M = Morphine, S = Saline.

Animals received additive doses of morphine twice a day for nine days. In order to induce withdrawal symptoms, naloxone (4 mg/kg, ip) was injected. The results in Figure 1 show that there was a significant (P < 0.001) difference in the TWS between saline + saline or DMSO 25% and control groups (morphine + saline or DMSO 25%). Data analysis indicated that the withdrawal signs including: jumping, rearing, genital grooming, abdomen writhing and wet dog shake were significantly greater in the control groups which received morphine compared to saline or DMSO 25% treated animals which means that the animals in the control group has already became dependent.

### Effect of icv administration of carbenoxolone on morphine withdrawal signs

**Figure 2:**
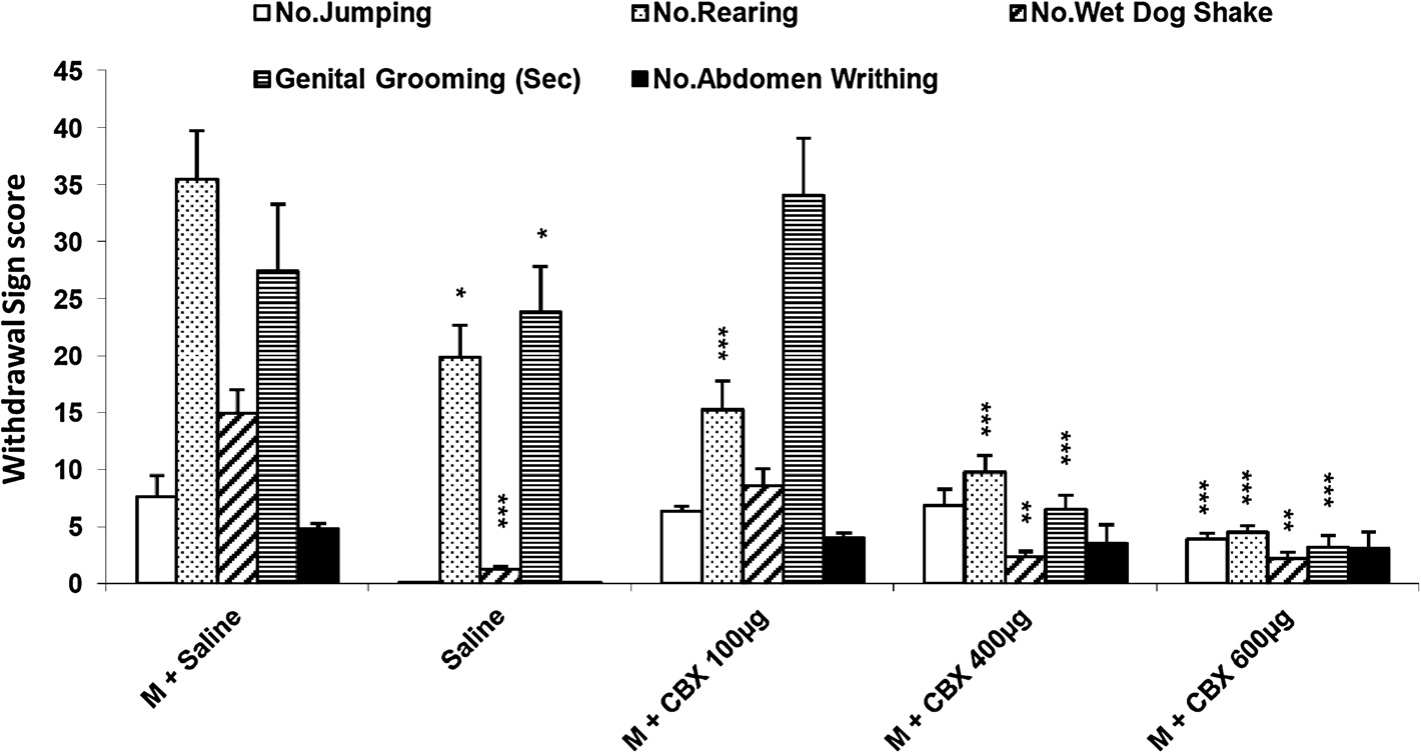
**Effects of icv injection of carbenoxolone (100, 400, 600 μg/rat) on the expression of naloxone (4 mg/kg)-induced withdrawal signs in morphine-dependent rats.** Data are expressed as mean ± S.E.M. ^*^p < 0.05, ^**^p < 0.01, ^***^p < 0.001 different from control (morphine-dependent DMSO 25% microinjected group). M = Morphine, S = Saline, CBX = carbenoxolone.

**Figure 3:**
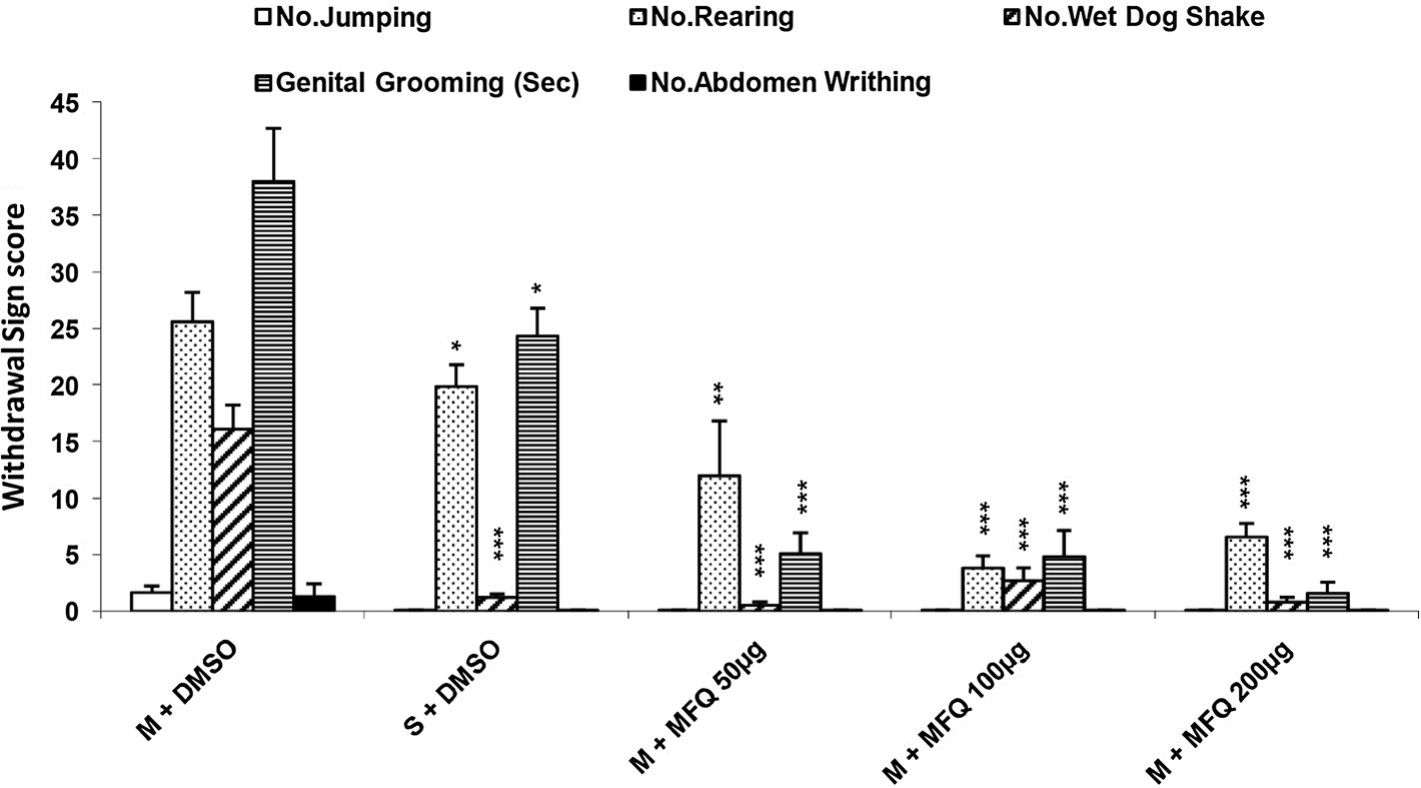
**Effects of icv injection of carbenoxolone (100, 400, 600 μg/rat) on the expression of naloxone (4 mg/kg)-induced TWS in morphine-dependent rats.** Data are expressed as mean ± S.E.M. ^***^p < 0.001 different from control (morphine-dependent DMSO 25% microinjected group). M = Morphine, S = Saline, CBX = carbenoxolone.

The results in Figure 2 revealed that icv injection of CBX (600 μg/10ul/rat, icv) significantly decreased all withdrawal signs except abdomen writhing [Figure 2] (P < 0.001 for all signs). In addition we found that carbenoxolone with all doses used in this study induced a significant decrease in TWS in comparison with the control group [Figure 3] (P < 0.001).

### Effect of icv administration of mefloquine on morphine withdrawal signs

**Figure 4:**
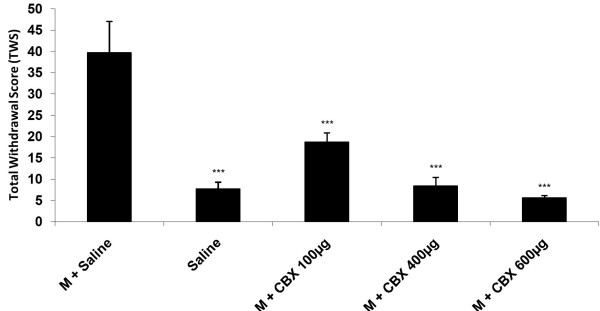
**Effects of icv injection of mefloquine (50, 100, 200 μg/rat) on the expression of naloxone (4 mg/kg)-induced withdrawal signs in morphine-dependent rats.** Data are expressed as mean ± S.E.M. ^*^p < 0.05, ^**^p < 0.01, ^***^p < 0.001 different from control (morphine-dependent DMSO 25% microinjected group). M = Morphine, S = Saline, MFQ = Mefloquine.

**Figure 5:**
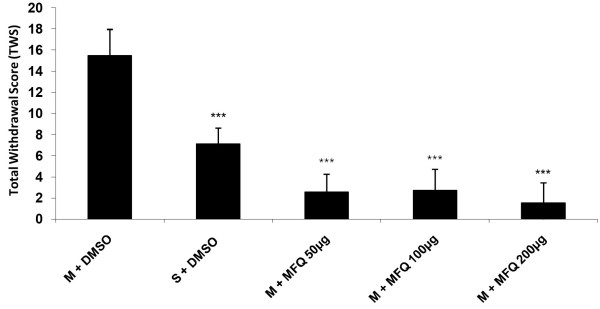
**Effects of icv injection of mefloquine (50, 100, 200 μg/rat) on the expression of naloxone (4 mg/kg)-induced TWS in morphine-dependent rats.** Data are expressed as mean ± S.E.M. ^***^p < 0.001 different from control (morphine-dependent DMSO 25% microinjected group). M = Morphine, S = Saline, MFQ = Mefloquine.

Our findings in this study have shown that icv administration of mefloquine (50, 100 and 200 μg/10 μl/ rat) decreased all withdrawal signs significantly [Figure 4] (P < 0.001). In addition the results indicated that icv injection of mefloquine resulted in a significant decrease in TWS compared to the control group [Figure 5] (P < 0.001).

The results of this study showed that stereotaxic surgery and icv injection of DMSO 25% had no effect on naloxone-induced opioid withdrawal symptoms in morphine-dependent rats while gap junction inhibitors (CBX and MFQ) significantly decreased the withdrawal signs. Locomotor activity test of rats with icv microinjected by CBX or MFQ was done to rule out the occurrence of motor performance hampering effect of drugs. The data showed that icv injection of CBX or MFQ had no significant effect on rats’motor performance. Thus, the alleviative effect of CBX or MFQ on opioid withdrawal is not ensued from suppression of motor activity.

Different drugs and strategies have been investigated to find an effective approach for preventing opioid dependence and withdrawal symptoms. Neurotransmitter systems, especially noradrenergic pathway has been found to play a key role in appearance of withdrawal symptoms. It has been reported that chronic morphine treatment may lead to a reduction in expression of inhibitory opioid peptides. These processes with the concurrently increase in the activity of glutamatergic neurons may lead to the enhancement of activity of noradrenergic system and somatic signs during drug dependence and withdrawal [30].

Some brain nucleuses especially LC appears to mediate somatic signs of opiate withdrawal. It has been reported that acute opiates administration inhibit LC neurons and subsequently inhibit cAMP This effect may lead to decrease in protein kinase A (PK_A_) activity and the phosphorylation rate of cAMP response element binding protein (CREB). These selective effects of morphine on cAMP protein phosphorylation indirectly generate specific behaviors commonly associated with addiction, tolerance and withdrawal [31,32]. Indeed if morphine treatment persists and is then abruptly terminated, LC neurons will increase the frequencies of action potentials. Therefore, the symptoms of morphine withdrawal will be produced [33,34].

Also it has been suggested that neuronal connections outside the LC play an important role in the withdrawal-induced activation of these nucleus [35].

The results of the present study for the first demonstrate that central administration of gap junction blocker such as carbenoxolone could alleviate opioid withdrawal symptoms in morphine-dependent rats.

Previous studies reported that CBX directly binds to and blocks a broad spectrum of the connexins that make up gap junctions [13,14]. CBX microinjected bilaterally into the substantia nigra (pars reticulata) also produced a dose-dependent reduction in the duration and severity of seizures [10].

CBX is a broad-spectrum gap junction blocker believed to act on a range of connexins and pannexins, with additional anti-inflammatory and mineralocorticoid- like properties [36], and has been shown in vitro to reduce seizure-like after discharges and spontaneous activity in electrical stimulation [37,38].

Experimental and theoretical evidence suggests that direct electrotonic communication between neurons via gap junctions, in combination with synaptic and ionic mechanisms, might contribute to the generation or maintenance of seizures [39-41].

Furthermore in a previous study, it has been shown that CBX might enhance the anticonvulsant action of some antiepileptics, such as diazepam, gabapentin, phenobarbital, felbamate and valproate [42], suggesting its potential usefulness in the human therapy of some types of pharmacoresistant epilepsies.

In another part of study we showed that MFQ as a potent and selective Cx36 gap junction blocker [43] could prevent opioid withdrawal signs and gathering the data from different symptoms according to Rasmussen et al. method, showed that mefloquine could attenuate the total withdrawal scores which is depicted in Figure 5.

Cx36 expression is found in almost all brain areas, including neocortex, brainstem, basal ganglia, hippocampus, and cerebellum [44,45]. Also MFQ is find to acts as an inhibitor of P-glycoprotein [46] and, therefore, it can increase the concentrations of other drugs in the brain [47].

In addition it has been shown that MFQ significantly increased IPSCs frequency in brain slices isolated from mouse [48] and this property might be helpful in attenuating of firings during withdrawal sings exhibition.

## Conclusion

Taking together we found that central administration of gap junction blockers; carbenoxolone or mefloquine prevented morphine-induced withdrawal symptom in rats.

## Competing interests

The authors declare that they have competing interests.

## Authors’ contributions

**SM**: contribution in doing the experiments. **MC**: contribution in study design. **HG**: contribution in data analysis and manuscript preparation. **RM**: contribution in doing the experiments. **MG**: contribution in doing the experiments and manuscript preparation. **KH**: contribution in study design, data analysis and manuscript preparation. All authors read and approved the final manuscript.
